# Global Disease Control in Inflammatory Arthritis Patients with Fibromyalgia Multi-Failure to Biologic Drugs: Short-Term Impact of Target Therapies on Both Disease Courses

**DOI:** 10.3390/jcm14196703

**Published:** 2025-09-23

**Authors:** Cinzia Rotondo, Silvia Stefania, Luigi Nardella, Ripalta Colia, Nicola Maruotti, Valeria Rella, Giuseppe Busto, Raffaele Barile, Francesco Paolo Cantatore, Addolorata Corrado

**Affiliations:** Rheumatology Unit, Department of Medical and Surgical Sciences, Azienda Ospedaliero-Universitaria Policlinico Riuniti di Foggia, Università degli Studi di Foggia, 71122 Foggia, Italyluigi_nardella.552809@unifg.it (L.N.); giuseppe.busto@unifg.it (G.B.);

**Keywords:** psoriatic arthritis, rheumatoid arthritis, fibromyalgia, biologic drugs, DMARDs, efficacy, retention rate, remission, low disease activity, pain improvement

## Abstract

**Background**: Fibromyalgia syndrome (FS) is one of the most common causes of chronic generalised pain and often complicates the therapeutic management of inflammatory chronic arthritis (ICA), negatively impacting both the real assessment of disease activity and the perception of response. Our study aims to evaluate in a group of patients with ICA, multi-resistant to biologic/target synthetic disease-modifying antirheumatic drugs (b/ts-DMARDs), both the impact of FS on the possibility of achieving low disease activity (LDA) or remission (REM) and the possible improvement in the severity of FS symptoms, after starting b/ts-DMARDs with different a mechanism of action (MoA). **Methods**: A prospective study was conducted, from January 2023 to December 2024, on patients who fulfil the classification criteria for psoriatic arthritis (PsA) or fulfil the 2010 American College of Rheumatology criteria for RA. **Results**: Sixty-four Caucasian patients with ICA, of which 47 with FS, were enrolled in the study. At the baseline visit, FS patients had a significantly shorter ICA disease duration, worse fibromyalgia symptom-related indices (such as Fibromyalgia Severity Scale (FSS), Widespread Pain Index (WPI), and Symptom Severity Scale (SSS)) and functional and disability scores (such as health assessment questionnaire (HAQ) and Functional Assessment of Chronic Illness Therapy (FACIT)), and a higher basal value of Disease Activity in Psoriatic Arthritis (DAPSA) score compared to patients without FS. After 6 months of starting b/ts-DMARDs, no differences in severity of arthritis clinimetric indices (disease activity score (DAS) 28 (erythrocyte sedimentation (ESR)) and DAPSA) and Visual Analogue Scale (VAS) pain were found between the patients with FS compared to those without. At the follow-up visit, 36% of the whole group of patients were in LDA (36% ICA patients with FS vs. 35% of ICA patients without FS; *p* = 0.080), while 17% of patients reached REM (11% ICA with FS vs. 35% ICA without FS patients; *p* = 0.031). The FS presence appeared to be a factor associated with failure to reach REM (OR 4.5 (95%CI: 1.1–17.8), *p* = 0.028), but not for achieving LDA (OR 2.7 (95%CI: 0.8–8.9), *p* = 0.099). The overall retention rate at 6 months was 79%; in particular, 11 patients discontinued treatment with b/ts-DMARD, 69% of whom belonged to the FS group (*p* = 0.489). Among the group of patients with ICA and FS, patients in LDA/REM presented an important improvement in FSS, SSS, and VAS pain, with the best percentage variation from the baseline of these indices compared to patients who did not achieve the LDA/REM. Of note, sixteen patients with FS at the baseline no longer met the diagnostic criteria for FS after 6 months of follow-up. **Conclusions**: The presence of FS seems to negatively impact the achievement of REM, but not LDA, in both RA and PsA patients, even in b/ts-DMARDs patients with multi-failure of at least two different MOAs. Only a cluster of patients with FS, presumably those with FS triggered and/or amplified by the chronic joint inflammatory process, appear to improve their perception of FS severity by achieving ICA LDA/REM. However, these findings require further supporting data for more accurate validation.

## 1. Introduction

Fibromyalgia syndrome (FS) is one of the most common causes of chronic generalised pain. It affects up to 5% of the general population [1]. It is characterised by a range of symptoms, including widespread pain associated with accompanying symptoms such as fatigue, sleep disturbances, cognitive dysfunction, depression, anxiety, bowel changes, and headache [1]. FS primarily affects women, with a 3:1 ratio, and typically occurs between the ages of 50 and 60 [2]. Although the true etiopathogenesis of FS is still unknown, clinical studies suggest a dysfunction of the central nervous system that leads to an increased transmission and perception of pain. Recently, research has increasingly focused on neuroinflammation and the possible autoimmune origin of FS. Essentially, FS is characterised by a reduced pain threshold, defined as hyperalgesia, and the perception of pain with innocuous stimuli, or allodynia [1,3].

FS often occurs concomitantly with other diseases that may act as triggers, or confounding and aggravating factors, such as rheumatoid arthritis (RA), spondyloarthritis (SpA), psoriatic arthritis (PsA), osteoarthritis, and thyroid disease [1]. The prevalence of FS in inflammatory arthropathies is considerably higher than in the general population: reaching 18–24% in RA, 14–16% in SpA, and 18% PsA. These data can be explained as a reflection of the high prevalence of fibromyalgia in the female gender, the latter being more frequently affected by RA than by seronegative arthritis [4]. Other factors related to the concomitant presence of FS in inflammatory arthropathies are a low level of education, high disease activity, and high enthesis score [5].

Many studies have highlighted how a significant percentage of patients with inflammatory chronic arthritis (ICA) do not respond or respond only partially to targeted therapies, identifying comorbidities, smoking, obesity, lifestyle, low socioeconomic status, poor adherence, pain syndromes, and fatty liver diseases as risk factors for difficult-to-treat patients [6,7]. Although it has been highlighted that the response to biologic disease-modifying antirheumatic drug (b-DMARD) therapies can also be influenced by serological and autoimmune factors [8,9,10,11,12,13,14,15,16], the main factors confounding the perception of the real state of the disease activity seem to be comorbidities, in particular, FS. Conflicting data have been published on ICA and FS patients, highlighting both the risk of overtreatment [17] and that of undertreatment [18]. However, the possibility of overestimating disease activity clinimetric scores, especially those containing patient-reported outcomes, in FS patients has been unanimously confirmed in all ICA, such as RA [18], PsA [19], and SpA [20].

Moreover, FS significantly complicates the evaluation of response to therapies, particularly b-DMARDs, in patients with ICA, interfering with patient-reported outcomes. Fibromyalgia-related symptoms, limitations of clinimetric indices, and neuroinflammation in FS may negatively impact the clinical evaluation of efficacy. Many studies have evidenced that it is important to consider FS in patients with ICA who do not achieve remission [21,22,23].

Although several data demonstrate the negative impact that FS has on the clinical assessment and therapeutic management of ICA, especially in the first line of b-DMARDs, very little data, exclusively performed on axial SpA patients, are available on how the symptoms and severity of FS may improve in ICA patients responding to target therapies [5,24]. So, it has been hypothesised that the association between high levels of inflammatory disease activity and the development of FS can be explained by the causal link between nociceptive stimulation and the development of central sensitisation [25]. Presumably, the reduction in inflammatory stimulus can improve the perception of chronic central pain, ameliorating the symptoms and severity of FS. Due to these bases, our study aims to evaluate in a group of patients with RA or PsA, multi-resistant to b/ts-DMARDs, both the impact of FS on the possibility of achieving low disease activity (LDA) or remission (REM) and the possible improvement in the severity of FS symptoms, after starting b/ts-DMARDs with different mechanisms of action (MoA).

## 2. Materials and Methods

### 2.1. Study Design and Patients

This prospective study was conducted on patients we followed at our outpatient clinic from January 2023 to December 2024. All patients enrolled were required to fulfil the classification criteria for psoriatic arthritis (PsA) [26] or fulfil the 2010 American College of Rheumatology criteria for RA [27]. Other inclusion criteria were as follows: age greater than or equal to 18 years, failure of at least 2 b/ts-DMARDs with different MoAs and started another b/ts-DMARDs with an MoA different from the previous ones at the enrolled visit (baseline visit) (patients on combined therapy with methotrexate and/or steroids could also be included), and stable therapy for the entire duration of the study. Previous diagnosis of FS was permitted, and the use of muscle-relaxants, antidepressants or anxiolytic drugs, commonly used in FS, was permitted in the case of stable therapy for almost 6 months preceding the enrollment time and for the entire duration of the study. The exclusion criteria were as follows: age less than 18 years old, inflammatory axial involvement, presence of inflammatory and/or autoimmune rheumatological diseases other than PsA and RA, neurological complications, autoimmune diseases, chronic kidney, liver diseases, pregnancy, inflammatory bowel disease, thyroid disorders, abuse or uncontrolled consumption of drugs, other chronic illnesses (such as cardiac and respiratory diseases), the presence of neuropathies or any other condition that can cause chronic neuropathic pain other than FS (diabetes mellitus, alcohol/drug abuse or dependence, history of recent infections, head or vertebral surgery, head or vertebral traumas, nerve compression diseases, malignancy or lymphoproliferative disease).

### 2.2. Clinical Assessment at the Baseline and Follow-Up Visit

All patients were administered the fibromyalgia severity score (FSS) test, composed of the SSS (Symptom Severity Scale) and WPI (Widespread Pain Index) at the baseline and follow-up visits. The FS diagnosis was established for WPI scores ≥ 7 and SSS ≥ 5, or WPI 4–6 and SSS ≥ 9 [28].

During each visit, physical, laboratory, instrumental, and clinical characteristics were assessed in all patients, including age, disease duration, current therapy, erythrocyte sedimentation rate (ESR), C-reactive protein (CRP), clinimetric indices (DAS 28 (ESR) in RA patients, DAPSA and LEI in patients with PsA), and patient-reported outcomes (VAS pain and FSS). Quality of life and disability were assessed using the Health Assessment Questionnaire (HAQ) and the FACIT Fatigue Scale.

Following the baseline visit, when a new b/ts-DMARD was started, each patient was reevaluated at 6 months.

### 2.3. Definition of Inflammatory Chronic Arthritis

For the purposes of the study, patients defined as having ICA include both PsA and RA diagnoses. We include just PsA and RA patients without axial involvement and exclude SpA patients to achieve homogeneity and similarity, considering just peripheral involvement, between patient groups.

### 2.4. Definition of Remission and Low Disease Activity

LDA and REM were established using DAS 28 (ESR) for RA patients and DAPSA for PsA patients.

### 2.5. Statistical Analysis

The results are presented as the mean ± S.D. (standard deviation) and as numerosity (percentage). The normal distribution was examined using the Shapiro–Wilk’s test. Student’s *t*-test evaluated comparisons between study groups of patients with FS and those without FS. The differences between categorical variables were realised by Pearson chi-square or Fisher’s exact test, as appropriate, followed by Bonferroni adjustment in the case of multiple comparisons. Univariate binary logistic regression analyses were used to evaluate the factors associated with reaching LDA or REM and with the loss of FS criteria at the 6th month of follow-up. Statistical significance was set at *p* ≤ 0.05. Statistical analysis was performed using IBM SPSS Statistics 26.

### 2.6. Ethics Approval

This study was approved by the local Ethics Committee (Ethics Review Board of Policlinico of Foggia, protocol number DDG 2623/2019—19 June 2019) and conducted in accordance with the ethical standards outlined in the 1964 Declaration of Helsinki and its subsequent amendments. All participants gave their informed consent to participate in this study.

## 3. Results

### 3.1. Main Demographic and Clinical Characteristics of the Patients

Sixty-four Caucasian patients met the study’s inclusion criteria, sixty were women and four were men. Fifty-three patients (83%) fulfilled the CASPAR criteria for PsA, and eleven patients (17%) satisfied the RA criteria. At the enrolled time, the mean age was 47 ± 10.1 years, while the mean disease duration was 10.4 ± 7.7 weeks. Forty-seven patients had a diagnosis of FS according to the FSS diagnostic criteria. The comparisons of clinical characteristics between ICA patients with FS and those without FS at the baseline visit and the 6-month follow-up visit are shown in Table 1 and Table 2, respectively. In addition, all selected patients had shown ineffectiveness to b/ts-DMARDs with at least two different MoAs.

Patients with FS had a significantly shorter ICA disease duration than those without FS (Table 1). Furthermore, FS patients had worse fibromyalgia symptom-related indices (such as FSS, WPI, and SSS), and functional and disability scores (as HAQ and FACIT) compared to patients without FS; they also used steroids more frequently, with higher doses (Table 1). Of note, the patients with PsA and FS had a higher basal value of DAPSA score (Table 1). No differences were found in the use of different mechanisms of action of b-DMARDs or JAK inhibitors, in the use of combination therapy with CS-DMARDs, in serum levels of ESR and CRP, and VAS pain between the two study groups (Table 1).

After 6 months of treatment, ICA with FS patients presented higher FSS, WPI, SSS, LEI, HAQ, and lower FACIT than those without FS (Table 2). Of note, no differences in severity of arthritis clinimetric indices (DAS 28 (ESR) and DAPSA), and VAS pain were found (Table 2).

### 3.2. Efficacy of b/ts-DAMRDs to Reach Low-Disease Activity or Remission at the 6-Month Follow-Up

At the 6-month follow-up visit, 36% of the whole group of patients were in LDA. In particular, 17 (36%) ICA patients with FS vs. 6 (35%) of ICA patients without FS were in LDA (*p* = 0.080), while just 11 (17%) patients reached REM, 5 (11%) ICA with FS vs. 6 (35%) ICA without FS patients (*p* = 0.031), specifically.

The FS presence appeared to be a factor associated with the failure to reach REM (OR 4.5 (95%CI: 1.1–17.8), *p* = 0.028), but not for achieving LDA (OR 2.7 (95%CI: 0.8–8.9), *p* = 0.099). Specifically, the basal values of SSS influenced the achievement of REM (OR 0.78 (95%CI: 0.63–0.97), *p* = 0.031) but not those of WPI (OR 0.87 (95%CI: 0.75–1.01), *p* = 0.069).

**Figure 1:**
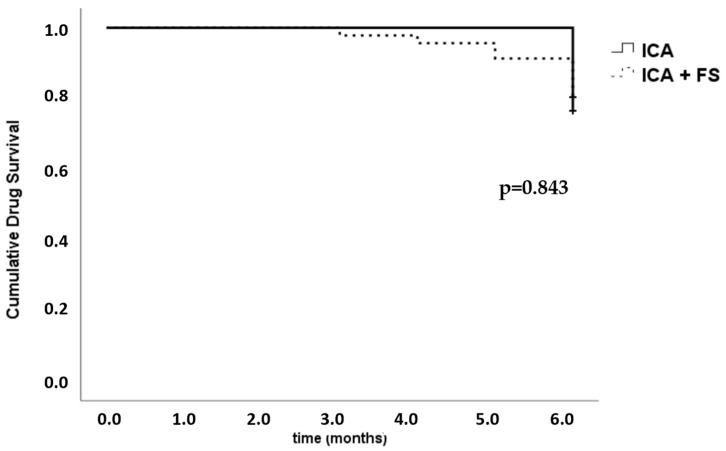
Kaplan–Meier survival function followed by the log-rank (Mantel–Cox) test. b/ts-DMARD survival in inflammatory chronic arthritis (ICA), divided based on the presence or absence of fibromyalgia (FS).

During the 6 months of the observational period, 11 patients discontinued treatment with b/ts-DMARD, 69% of whom belonged to the FS group (*p* = 0.489). The overall retention rate at 6 months was 79%. No significant differences were found in b/ts-DMARD survival between the ICA groups of patients with or without FS (Figure 1).

To evaluate the possible improvement of the pain component, the severity of FS, the symptoms related to FS, and the main clinical differences between the group of patients who achieved LDA or REM compared to those who did not achieve them, we considered only the group of patients with ICA and FS. The principal characteristics of both groups of patients in LDA/REM and those in high/moderate disease activity (no LDA/REM) are reported in Table 3. The group of patients in LDA/REM presented an important improvement in FSS, SSS, and VAS pain, with the best percentage variation from the baseline of these indices (Table 3). LDA and REM patients used fewer steroids (Table 3). Of note, the disease activity status was defined based on DAS 28 (ESR) for RA patients and DAPSA for PsA patients.

No differences were observed in patients in various mechanisms of action and different lines of b/ts-DMARD treatment.

### 3.3. Impact on Fibromyalgia Syndrome

The sixteen patients (32%) with FS at the baseline no longer met the diagnostic criteria for FS after 6 months of follow-up. Table 4 shows the main clinical characteristics of ICA patients with FS at the baseline who continued to meet the FS criteria after 6 months, compared to those who no longer met them. We underscore that in patients who no longer meet the FS criteria, a significant percentage improvement from the baseline was observed in all FS indices (such as FSS, WPI, and SSS), and the perception of disability (HAQ) compared to ICA patients who continue to meet FS criteria, while no significant differences were observed in arthritis clinimetric score (Table 4). The b/ts-DMARD retention rate at the 6-month follow-up is higher in ICA patients who did not meet the FS criteria (86%) compared to those who continue to meet the FS criteria.

No differences were found between the two groups in all ICA and FS clinimetric indices at the baseline visit. Of note among steroid users, 83% were the FS patients who persisted to fulfil FS criteria at the 6-month follow-up visit.

Among the variables studied, through binary logistic regression analysis, none was found to be able to predict the improvement of FS scores.

## 4. Discussion

In the present study, which shows real-life data of patients with RA and PsA resistant to at least two b/ts-DMARDs with different mechanisms of action, we mainly found that the presence of FS can interfere with the achievement of REM, but not with the achievement of LDA, with a trend towards a lower retention rate in a 6-month observation period. These data partially echoed previous studies, showing that FS could negatively influence the response to b-DMARDs, evidencing the presence of high disease activity scores during treatment [29], a lower retention rate, and a greater switching between anti-TNF (in patients with axial SpA) [5] and a lower retention rate of REM/LDA in PsA patients [21,23]. So, according to these studies, FS seems to negatively impact the achievement of a good clinical response in ICA patients. Furthermore, in our study, the presence of FS, and, in particular, high SSS values at the baseline, but not WPI, seems to predict failure to reach REM but not LDA. This latter finding about SSS has been previously described only in axial SpA but not in RA and PsA [24].

Another important issue, widely discussed in already published studies, on FS as a comorbidity of ICA, is the negative impact on the real state of disease activity [30,31,32]. Indeed, there are several symptoms common to FS and ICA. In our study, we found that patients with FS presented more severe scores of DAPSA and a higher trend for DAS 28 (ESR) at the baseline visit. This difference disappears in the first 6 months of follow-up, which is in line with many studies showing that disease activity and disability indices are higher in patients with arthritis without FS, both in RA and PsA [19,33,34]. Furthermore, a study on RA showed that DAS28 and HAQ were higher in patients with fibromyalgia and arthritis than in patients with arthritis alone at the baseline, while this difference tended to disappear with continued follow-up [18]. Another study on axSpA showed higher BASDAI values at the start of the anti-TNF treatment in patients with FS, while at 12 months of treatment, the value was comparable [5]. Partially contrasting with published findings [5,18], in the present study, we found different results for LEI, HAQ and FACIT, which, although are improving compared to the baseline visit, remain significantly worse than patients without FS at the sixth-month visit. In addition to a greater perception of disability and fatigue, probably due to the presence of chronic pain associated with FS, the data on LEI could be explained by the fact that the location of the tender points is very similar to that of the entheses, and, therefore, it may be difficult to distinguish enthesitis from fibromyalgia pain.

While there is evidence in the literature showing the negative impact that FS has on the therapeutic management of ICA, some studies have demonstrated improvement in FS symptoms in arthritis patients treated with b-DMARDs. Macfarlane et al. showed that treatment with anti-TNF drugs in patients with axial SpA led to an improvement in FS symptoms at 12 weeks. Specifically, the study showed that with the improvement of the disease, three of the five patients with FS at the baseline no longer met the 2011 fibromyalgia criteria at follow-up, thanks to an improvement in both WPI and SSS [24]. Molto et al. also showed how the percentage of FS decreased over time in patients treated with anti-TNF [5]. In line with these data, in our study, we observed that 32% of patients with FS, at the baseline visit, improved their FS-related symptoms so much that they no longer met the FS criteria after 6 months of observation. But even more importantly, for the first time, we found that patients with ICA and FS who reach LDA or REM at 6 months showed a significant percentage of improvement in all FS scores (such as FSS, WPI and SSS). This finding has never been described in previous studies. The important role of neuroinflammation in the pathogenesis of FS, supported by the detection of elevated levels of pro-inflammatory cytokines (such as IL-8, IL-6, TNFα, IL-17, IL-1β, and leptin) in serum and cerebrospinal fluid and the finding of high frequencies of FS in ICA, may lead to the hypothesis that peripheral inflammation increases peripheral nociceptive input and causes chronic pain through central sensitisation [22,35]. Other investigations also evidenced, together with an increase in pro-inflammatory cytokines, a reduction in anti-inflammatory cytokines (such as IL-4 and IL-13) [36,37]. This imbalance determines immune dysregulation, evidenced by increased neutrophil/lymphocyte ratio, and T lymphocyte subpopulation abnormalities, particularly in CD4+ T-cells and natural killer T-cells [38,39]. So, the association between peripheral inflammation and FS could go beyond the simple pain perception and amplification [4,40], but underlying this, uncontrolled inflammatory pathways may likely trigger a self-sustaining mechanism in which pain, inflammation and immune imbalance amplify each other [41]. These hypotheses should support our findings. Moreover, considering that not all patients with FS improve their perception of FS symptoms, it could be hypothesised that there may be different subsets of FS, one triggered or worsened by the presence of systemic inflammation associated with ICA and another subset of FS totally independent from it. But this fascinating hypothesis cannot be proven as we have not found any predictors of a better response among the variables considered in this study. Furthermore, the lack of significant differences in clinical variables and ICA clinimetric scores between the group of patients who continue to meet FS criteria after 6 months and those who no longer meet them may be due to the small sample size. However, a trend toward improved percentage change from the baseline for almost all scores was observed.

The choice to conduct the study on a particular cluster of ICA patients, such as the multi-resistant one, was dictated by the necessity to evaluate the possibility of clinical response to b/ts-DMARDs with a different MoA in patients with longer pain chronicity, as well as to provide real-life data that were previously missing.

In regard to the matter of overtreatment in FS patients with ICA, we underline that higher rates of steroid users were found among FS patients, namely those who persisted in fulfilling the FS criteria at the 6-month follow-up visit, and those who did not achieve REM or LDA in the observational period. Of note, the use of steroids at very low doses, suggested for a short period in RA treatment recommendations, but not in recommendations for PsA patients, may be due firstly to long-standing disease duration (>5 years), self-administration at the onset of disease, and then to steroid-dependence of patients for pain relief. Furthermore, the significant difference in the duration of ICA disease, which is shorter in patients with FS, confirms the evidence widely described in the literature on the clinical risk of tending to anticipate the initiation of b/ts-DMARDs in patients with FS.

This study has some limitations. The first one is the small sample size, primarily due to the strict inclusion criteria. Another limitation is the heterogeneity of b/ts-DMARDs initiated at enrollment, but one of the aims of our study was to evaluate whether good ICA control could modify pain perception and FM scores, and not to evaluate whether one b-DMARD’s MoA was more effective than others. Some limitations may arise from the actual assessment of LEI. To assess whether FS could be a confounding factor by overestimating LEI, at least an ultrasound assessment would have been necessary, but this was not performed in this study. Furthermore, the exclusion of SpA patients, namely those with axial involvement and other types of ICA, may limit the generalizability of the result, but allow the homogenisation of pure peripheral patterns. Lastly, assessing the presence and severity of FS using only the 2016 ACR criteria [28] could be biassed. However, for clinimetric assessment purposes, the 2016 ACR criteria [28] appear to be the most effective.

## 5. Conclusions

The presence of FS appears to negatively impact the achievement of REM, but not LDA, in both RA and PsA patients, even in b/ts-DMARD patients with multi-failure of at least two different MOAs. A discrete percentage of responses to another MoA can be expected in this class of difficult-to-treat patients. Due to the observed improvement in FS severity in patients achieving LDA or REM, it is possible to hypothesise the presence of different clusters of secondary FS. Indeed, just the FS patients who achieved LDA/REM, presumably those with FS triggered and/or amplified by the chronic joint inflammatory process, appear to improve their perception of FS severity. However, these findings require further supporting data for more accurate validation.

## Figures and Tables

**Table 1:** Comparison of demographic and clinical characteristics at the baseline visit between the group of inflammatory chronic arthritis (ICA) patients with fibromyalgia syndrome (FS) and those without FS.

	ICA + FS *n* = 47	ICA *n* = 17	*p*-Value
Age (years)	46.7 ± 11.5	47.8 ± 8.1	0.724
Female/Male	45/2	15/2	0.285
BMI (kg/m^2^)	27.4 ± 5.5	27.5 ± 4.9	0.931
Diagnosis			
RA	6 (13)	5 (29)	0.295
PsA	41 (87)	12 (71)	0.325
**Disease duration (years)**	**9 ± 6.8**	**14.5 ± 8.7**	**0.011**
DAS 28 (ESR)	4.1 ± 0.8	3.7 ± 1.1	0.216
**DAPSA**	**26.3 ± 7.0**	**19.1 ± 7.2**	**0.004**
ERS (mm/h)	32.6 ± 14.8	28.8 ± 16.7	0.275
CRP (mg/L)	6.2 ±4.8	4.6 ± 3.9	0.254
LEI	1.5 ± 1.4	1.3 ± 1.4	0.711
**FSS**	**20.1 ± 3.8**	**7.1 ± 3.6**	**0.0001**
**WPI**	**11.0 ± 3.9**	**2.8 ± 2.4**	**0.0001**
**SSS**	**8.9 ± 2.1**	**4.2 ± 2.4**	**0.0001**
VAS pain	73.0 ± 16.7	60.1 ± 25.5	0.065
**FACIT**	**23.3 ± 9.6**	**36.6 ± 10.4**	**0.0001**
**HAQ**	**1.3 ± 0.8**	**0.7 ± 0.6**	**0.016**
25-hydroxyvitamin D (ng/mL)	25.3 ± 12.1	42.9 ± 23.9	0.088
MoA of b/ts-DMARDs started			
Anti-TNFα	22 (47)	6 (35)	0.698
No anti-TNFα	19 (40)	8 (47)	0.696
Abatacept	1 (2)	1 (6)	0.341
Anti-IL-23	5 (11)	2 (11)	0.964
Anti IL-17	10 (21)	3 (18)	0.842
Anti IL-6	3 (6)	2 (11)	0.639
JAK-inhibitors	6 (13)	3 (18)	0.412
**Steroid users**	**20 (43)**	**2 (12)**	**0.019**
**Equivalent prednisone dose (mg)**	**2.2 ± 2.8**	**0.2 ± 0.8**	**0.008**
Combo therapy with MTX	18 (39)	9 (53)	0.223

DAPSA: Disease Activity in Psoriatic Arthritis. DAS: disease activity score. FACIT: Functional Assessment of Chronic Illness Therapy. ESR: Erythrocyte sedimentation. FSS: Fibromyalgia Severity Scale. HAQ: Health Assessment Questionnaire. LEI: Leeds Enthesitis Index. SSS: Symptom Severity Scale. VAS: Visual Analogue Scale. WPI: Widespread Pain Index.

**Table 2:** Comparison of clinimetric scores at 6-month follow-up visit between ICA patient groups with FS and those without.

	ICA + FS	ICA	*p*-Value
DAS 28 (ESR)	3.1 ± 1	2.6 ± 0.8	0.088
DAPSA	17.5 ± 10.4	13.0 ± 8.8	0.114
**LEI**	**0.9 ± 1.1**	**0.2 ± 0.6**	**0.045**
**FSS**	**16.1 ± 7.4**	**7.6 ± 4.6**	**0.0001**
**WPI**	**8.7 ± 5.5**	**3.7 ± 3.1**	**0.001**
**SSS**	**7.3 ± 3.3**	**3.9 ± 2.3**	**0.0001**
VAS pain	54.2 ± 29.8	42.6 ± 23.8	0.154
**FACIT**	**27.3 ± 10.8**	**38.7 ± 9.2**	**0.0001**
**HAQ**	**1.1 ± 0.6**	**0.4 ± 0.4**	**0.0001**

DAPSA: Disease Activity in Psoriatic Arthritis. DAS: disease activity score. FACIT: Functional Assessment of Chronic Illness Therapy. ESR: Erythrocyte sedimentation. FSS: Fibromyalgia Severity Scale. HAQ: Health Assessment Questionnaire. LEI: Leeds Enthesitis Index. SSS: Symptom Severity Scale. VAS: Visual Analogue Scale. WPI: Widespread Pain Index.

**Table 3:** Comparison of clinical characteristics between inflammatory chronic arthritis (ICA) patients with fibromyalgia syndrome (FS) who reached low disease activity (LDA) or remission (REM) at the 6-month follow-up visit and those who did not achieve them. All scores are expressed as percentage variation (Δ%) from the baseline.

	LDA/REM	No LDA/REM	*p*-Value
**Δ%FSS**	**31.0 ± 32.9**	**8.4 ± 37.7**	**0.034**
Δ%WPI	31.9 ± 48.9	4.5 ± 57.3	0.087
**Δ%SSS**	**28.6 ± 32.4**	**−0.9 ± 52.9**	**0.028**
**Δ%VAS pain**	**54.4 ± 38.4**	**4.5 ± 20.1**	**0.0001**
Δ%FACIT	−40.0 ± 58.0	−17.0 ± 53.3	0.172
Δ%HAQ	13.1 ± 64.1	2.0 ± 53.5	0.537
Diagnosis			
RA	2 (9)	4 (16)	0.479
PsA	20 (91)	21 (84)	0.397
**Steroid users**	**5 (21)**	**14 (54)**	**0.017**
Failure to satisfy FS criteria at 6-month follow-up visit	9 (38)	5 (19)	0.151

FACIT: Functional Assessment of Chronic Illness Therapy. FSS: Fibromyalgia Severity Scale. HAQ: Health Assessment Questionnaire. LDA: low-disease activity. PsA: psoriatic arthritis. RA: rheumatoid arthritis. SSS: Symptom Severity Scale. VAS: Visual Analogue Scale. WPI: Widespread Pain Index.

**Table 4:** Comparison of clinical characteristics between ICA patients with FS at the baseline visit, divided based on whether (FS persistence) or not (no more FS) FS diagnostic criteria were met at the 6-month follow-up. All scores are expressed as percentage variation (Δ%) from the baseline.

	No More FS	FS Persistence	*p*-Value
Δ% DAS 28 (ESR)	21.3 ± 23.3	24.3 ± 29	0.742
Δ% DAPSA	48.5 ± 31.6	29.4 ± 35.9	0.107
Δ% LEI	61.1 ± 48.5	28.1 ± 54.6	0.146
**Δ% FSS**	**56.8 ± 15.6**	**−3.1 ± 27.7**	**0.0001**
**Δ% WPI**	**65.0 ± 23.3**	**−11.4 ± 52.7**	**0.0001**
**Δ% SSS**	**48.5 ± 26.2**	**−8.1 ± 40.1**	**0.0001**
Δ% VAS pain	41.8 ± 37.2	22.9 ± 37.3	0.135
Δ% FACIT fatigue scale	−51.3 ± 77.0	−16.9 ± 40.4	0.064
**Δ% HAQ**	**43.6 ± 40.4**	**−6.4 ± 61.9**	**0.018**
LDA	6 (50)	11 (35)	0.291
Remission	2 (17)	2 (6)	0.121
b/ts-DMARDs retention rate	12 (86)	27 (77)	0.403

DAPSA: Disease Activity in Psoriatic Arthritis. DAS: disease activity score. DMARDs: Disease-modifying antirheumatic drugs. ESR: Erythrocyte sedimentation. FACIT: Functional Assessment of Chronic Illness Therapy. FSS: Fibromyalgia Severity Scale. HAQ: Health Assessment Questionnaire. LDA: low-disease activity. LEI: Leeds Enthesitis Index. SSS: Symptom Severity Scale. VAS: Visual Analogue Scale. WPI: Widespread Pain Index.

## Data Availability

The data are part of an ongoing study and cannot be published.

## Abbreviations

BMI	Body Mass Index
DAPSA	Disease Activity in Psoriatic Arthritis
DAS	disease activity score
DMARDs	Disease-modifying antirheumatic drugs
ESR	Erythrocyte sedimentation
FACIT	Functional Assessment of Chronic Illness Therapy
FSS	Fibromyalgia Severity Scale
HAQ	Health Assessment Questionnaire
ICA	inflammatory chronic arthritis
LDA	low-disease activity
LEI	Leeds Enthesitis Index
MoA	mechanism of action
MTX	Methotrexate
PsA	psoriatic arthritis
RA	rheumatoid arthritis
SSS	Symptom Severity Scale
TNF	Tumour Necrosis Factor
VAS	Visual Analogue Scale
WPI	Widespread Pain Index
